# Methotrexate and a spleen tyrosine kinase inhibitor cooperate to inhibit responses to peripheral blood B cells in rheumatoid arthritis

**DOI:** 10.1002/prp2.16

**Published:** 2013-12-15

**Authors:** Greg Coffey, Andreas Betz, Jonathan Graf, Gillian Stephens, Pei Hua Lin, John Imboden, Uma Sinha

**Affiliations:** 1 Portola Pharmaceuticals, Inc.South San Francisco, California, 94080; 2 University of California San Francisco, School of MedicineSan Francisco, California, 94143

**Keywords:** B cells, methotrexate, rheumatoid arthritis, spleen tyrosine kinase

## Abstract

**Background:**

Selective disruption of the spleen tyrosine kinase (Syk) represents a novel strategy to control B-cell functional responses by inhibition of B-cell antigen receptor (BCR) signaling. PRT062607 (P505-15) is a highly selective small molecule Syk inhibitor that potently suppresses B-cell function in human and rodent blood, and reduces inflammation in rodent models of rheumatoid arthritis (RA).

**Aims:**

In this study, we sought to determine the potency of Syk inhibition by PRT062607 in whole blood from RA patients, and elucidate covariates that affect the potency of immune-regulation by this compound.

**Materials and Methods:**

Whole blood was collected from 30 patients diagnosed with RA as part of a single-center outpatient study. Disease severity, serum protein markers of inflammation, and co-medications were related to each other, and to PRT062607 activity in ex vivo Syk-mediated immune function assays.

**Results:**

We report here that PRT062607 exhibited greater potency in suppressing BCR mediated B-cell functional responses in whole blood from RA patients who received stable methotrexate (MTX) therapy. We demonstrate that the B-cell functional response to BCR ligation is influenced by cytokines and JAK/STAT signaling.

**Discussion:**

MTX is a known cytokine modulating agent, and this mechanism may act in concert with PRT062607 to control B-cell function.

**Conclusion:**

These data have important implications for the co-administration of Syk inhibitors and MTX for the treatment of RA.

## Introduction

MTX is widely used to control aberrant immune function in a variety of diseases. One mechanism by which MTX may suppress immune function is by reducing proinflammatory cytokine burden via increasing extracellular concentrations of adenosine (reviewed by [Wessels et al. 2008]). Adenosine engages the A2a/b adenosine receptor expressed on various cell types initiating a signaling pathway that leads to suppression of cytokine signaling and inhibits NFkB. Consequently, cells are rendered less responsive to cytokines, and have a diminished capacity to produce cytokines (Cutolo et al. 2001). Thus, adenosine levels are elevated in animals treated with MTX, and immune suppression resulting from MTX treatment is blocked by adenosine receptor antagonism (Cronstein et al. 1993). Adenosine and the AICAR metabolite aminoimidazolecarboxamide are also elevated in patients treated with MTX (Baggott et al. 1999; Riksen et al. 2006), and the therapy is directly associated with decreased serum levels of various cytokines, including tumor necrosis factor α (TNF), interferon γ, IL6, IL8, IL10, IL12, and macrophage inflammatory protein 1α (Chan and Cronstein 2002; Kraan et al. 2004). Treatment of peripheral blood mononuclear cells with MTX significantly reduced the cell's capacity to synthesize IL2 and interferon γ mRNA in response to phytohemagglutinin (Constantin et al. 1998). Hence, MTX has been demonstrated in both animal models and in patients to be a potent cytokine modulating agent.

We recently reported on the activity of PRT062607 (also called P505-15), a selective and potent inhibitor of Syk that elicits anti-inflammatory activity in rodent models of RA (Coffey et al. 2011). PRT062607 suppresses signaling downstream of the B cell antigen receptor (BCR) and fragment crystallizable epsilon receptor I (FcεRI), and consequently inhibits B cell and basophil functional responses. Importantly, however, B-cell function is regulated by several costimulatory factors that operate independent of the BCR/Syk complex. Several cytokines in particular are reported to prime or potentiate B-cell responses to BCR engagement, including interferon α/β, IL2, and IL4 (Tsudo et al. 1984; Waldmann et al. 1984; Zubler et al. 1984; Muraguchi et al. 1985; Clark et al. 1989; Butcher and Cushley 1991; Braun et al. 2002). Similarly, the threshold for FcεRI-mediated basophil degranulation is lowered by costimulation with IL3. Therefore, cytokine reduction therapies may have a potentiating effect on the expected inhibition of Syk-dependent immune functional responses.

In this study, we evaluated the impact of disease severity, serum protein markers of inflammation, and concomitant medications on the potency of PRT062607 in B-cell and basophil functional assays using whole blood from RA patients. We report here that patients with severe disease presented with reduced PRT062607 potency in a whole blood assay measuring BCR-mediated B-cell activation, a phenomenon that was corrected in patients receiving stable MTX therapy. MTX diminished the B cells' ability to functionally respond to BCR ligation, but did not influence BCR/Syk signaling or FcεRI/Syk-mediated basophil degranulation. These data suggested that MTX operated via a mechanism independent of Syk to control BCR-mediated B-cell activation. To explore this further, we found that patients on stable MTX therapy, irrespective of disease severity, had reduced serum cytokine levels, including IL2, a known costimulatory factor for B-cell activation. Costimulation with IL2 (a JAK1/3-dependent pathway) significantly enhanced BCR-mediated CD69 upregulation by B cells, and subtly but significantly affected the potency of PRT062607 in suppressing this functional response. Furthermore, combined Syk-selective and JAK-selective small molecule kinase inhibitors were significantly more effective at inhibiting BCR-mediated B-cell activation relative to either inhibitor alone. We conclude from these studies that B-cell functional responses are influenced by both BCR/Syk and cytokine/JAK-dependent signaling pathways. Furthermore, MTX may cooperate with Syk inhibition to control B-cell functional responses by reducing cytokine burden.

## Materials and Methods

### Study design and patient enrollment

Peripheral blood samples were obtained after written consent from 30 male and female patients (detailed in Table 1) who were recruited from the RA Clinic at San Francisco General Hospital. Patients had to fulfill the 1987 American College of Rheumatology Classification Criteria for RA, be between the ages of 18 and 80 years, and be able to give informed consent. Disease Activity Score 28 joints (DAS28) was determined using the patient global assessment, tender and swollen joint counts (by an attending rheumatologist), and C-reactive protein (CRP) and erythrocyte sedimentation rate (ESR) measured on the day of phlebotomy. DAS scores were defined as Remission (<2.6), Mild (≥2.6 to <3.2), Moderate (≥3.2 to <5.1), and Severe (≥5.1). This study was approved by the Committee for Human Research of the University of California San Francisco (the Institutional Review Board), and was carried out in accordance with the Declaration of Helsinki.

**Table 1:** Baseline demographics and patient characteristics

Patient demographic			Patient characteristics						Concomitant medications				
Subject	Gender	Race	TJC	SWC	ESR	CRP	DAS28ESR	DAS28CRP	RF	CCP	MTX	Pred.	TNF Inh.
1	F	Asian	9	10	15	0.7	5.21	4.47	Neg	Pos	Yes	Yes	No
2	F	White	1	17	35	5.3	4.03	3.16	Pos	Pos	Yes	Yes	No
3	F	Asian	8	5	44	30.4	6.04	5.59	Pos	Pos	No	Yes	Yes
4	F	Asian	2	16	21	1.3	4.18	3.31	Pos	Pos	Yes	Yes	No
5	F	Asian	9	3	17	1.2	5.07	4.33	Pos	Pos	No	Yes	No
6	F	Asian	1	14	50	11.4	4.1	3.22	Neg	Pos	No	No	No
7	F	White	0	1	30	7	2.44	1.76	Pos	Neg	Yes	Yes	No
8	F	White	2	2	9	3.4	2.96	2.92	Neg	Neg	Yes	No	Yes
9	F	White	3	4	24	2.3	4.18	3.34	Pos	Pos	Yes	Yes	Yes
10	F	White	1	14	10	15.1	3.43	3.78	Pos	Neg	Yes	NO	NO
11	F	White	13	5	66	34.4	6.93	6.24	Pos	Pos	Yes	Yes	No
12	F	White	9	3	41	5.6	6.2	5.24	Neg	Neg	Yes	Yes	Yes
13	F	White	0	2	35	27.2	2.85	2.53	Pos	Pos	No	Yes	Yes
14	F	White	1	11	12	0.8	2.75	2.18	Pos	Pos	Yes	No	No
15	F	White	7	3	51	9.4	6.28	5.33	Pos	Pos	Yes	Yes	No
16	F	Asian	0	2	70	30.8	3.8	3.03	Pos	Pos	Yes	Yes	NO
17	F	White	8	11	27	7.4	4.54	3.96	Pos	Neg	Yes	No	No
18	F	White	6	9	20	8.2	5.83	5.49	Pos	Pos	No	Yes	Yes
19	F	White	14	12	77	45.6	7.61	6.91	Pos	Pos	No	Yes	No
20	F	White	1	2	14	13.2	3.53	3.6	Pos	Pos	No	Yes	No
21	F	White	0	1	10	1.2	1.64	1.27	Pos	Pos	Yes	Yes	Yes
22	F	Asian	7	8	28	19.4	5.62	5.33	Pos	Pos	Yes	No	No
23	F	White	4	15	25	1.6	4.62	3.67	Pos	Pos	No	Yes	No
24	F	White	0	11	13	1	2.43	1.84	Neg	Neg	Yes	Yes	No
25	F	Asian	8	6	19	2.5	5.34	5	Pos	Pos	No	Yes	No
26	F	White	5	7	17	2.3	4.86	4.27	Pos	Pos	No	Yes	No
27	M	Asian	3	14	26	3.6	4.2	3.43	Pos	Pos	Yes	Yes	Yes
28	F	White	2	8	18	6.6	4.76	4.43	Pos	Pos	No	Yes	No
29	F	White	2	14	29	2.6	3.77	2.83	Pos	Pos	No	No	Yes
30	F	Asian	1	14	24	1.2	3.46	2.48	Pos	Pos	No	Yes	No
31	M	White	18	13	35	3.3	6.75	5.75	Pos	Pos	No	Yes	No
32	F	White	0	2	15	1.1	2.5	1.83	Pos	Pos	Yes	No	Yes

Various measures of disease activity and concomitant mediations are detailed for each patient. CCP, citrullinated protein; CRP, C-reactive protein (pg/mL); DAS28CRP, disease activity score 28-CRP; DAS28ESR, disease activity score 28-ESR; ESR, erythrocyte sedimentation rate; MTX, methotrexate; Pred, prednisone; RF, rheumatoid factor; SWC, swollen joint count; TJC, tender joint count; TNFInh, tumor necrosis factor inhibitor.

### Reagents

Sodium heparin vacutainer tubes (4 mL) were obtained from BD Diagnostics (Franklin Lakes, NJ). The BasoTest kit was obtained from Orpegen Pharma (Heidelberg, Germany). Antibodies used in these studies were anti-human IgE and IgD (Bethyl Laboratories, Montgomery, TX), anti-human Erk Tyr204 (Cell Signaling Technologies, Danvers, MA), anti-human CD19 peridinin chlorophyll and allophycocyanin-conjugated, anti-human CD69 phycoerythrin-conjugated, and anti-human Syk Tyr352 phycoerythrin-conjugated (BD Biosience, San Jose, CA). Goat anti-rabbit allophycocyanin-conjugated antibody was obtained from Jackson Immunoresearch (Westgrove, PA). Cytokines utilized were IL2 and IL4 (R&D Systems, Minneapolis, MN). Fluorescence-activated cell sorting/lyse solution was obtained from BD Bioscience.

### Human whole blood ex vivo immune function tests

Blood from healthy donors was collected from the antecubital vein into 4 mL sodium heparin vacutainer tubes from subjects who gave written informed consent to the protocol (approved by the Human Subjects Committee of Portola Pharmaceuticals Inc.). Blood obtained from healthy donors or patients with RA was then used for ex vivo induction of basophil degranulation, B-cell activation, and B-cell receptor signaling, as described elsewhere (Coffey et al. 2011). Briefly, 100 μL aliquots of blood were pretreated for 30 min with various concentrations of PRT062607 or CP690,550 (specific JAK1/3 inhibitor; tofacitinib), as indicated in the Results. Following this time, blood was stimulated with anti-IgE (1:333 dilution in PBS) for 20 min to induce FcεRI-mediated basophil degranulation. This was measured by CD63 upregulation on the cell surface using the BasoTest kit and supplied protocol. Blood was also stimulated for 16 h with anti-IgD to induce BCR-mediated B-cell activation, and then stained for upregulation of the early activation marker CD69. To measure BCR-induced signaling events, blood was stimulated for 10 min with anti-IgD, after which cells were fixed and permeabilized and stained for Erk Tyr204 and Syk Tyr352. In some experiments, blood aliquots were mixed with 10 ng/mL IL2 or IL4 immediately prior to inclusion of anti-IgD to determine the impact of cytokine costimulation on B-cell function and reliance on Syk following BCR ligation. Flow cytometry was performed using the FACS Calibur (BD Biosciences), in which 2000 events were collected for the relevant cell populations. Mean fluorescent intensities were quantified using FlowJo software (Tree Star Inc., Ashland, OR).

### Serum protein quantitation

Whole blood (10 mL) was collected for preparation of serum, which was immediately aliquoted and snap frozen on a mixture of dry ice and methanol. Samples were stored at −80°C prior to analysis by Ricerca Biosciences (Concord, OH) for concentrations of various cytokines and other serum protein markers of disease activity in RA. Samples were thawed on ice and aliquoted into a 96-well PCR plate. Serum was diluted 1:100 for serum amyloid A1 and leptin analysis, and 1:10 for matrix metalloproteinase-1 and matrix metalloproteinase-3 analysis. Serum proteins were quantified using Luminex technology (Wong et al. 2008).

### Statistical analysis

The R programming environment was used for data analysis and graphics. The dose-response curves of inhibition were analyzed by nonlinear regression to the logistic curve using the following equation (Ritz 2005).

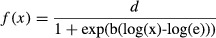


The parameter b represents the slope and e the concentration at half inhibition (IC50). The parameter d was set to 100, consistent with complete inhibition. The approximate confidence intervals for the IC50 were calculated by serial expansion using the delta method. The correlation of the biomarkers in serum with the DAS28 CRP and DAS28 ESR was quantified by the Pearson correlation coefficient and the values are illustrated in a heat map. For pairwise comparisons between populations the Wilcoxon test at a confidence level alpha = 0.05 was used with a correction for ties resulting from detection limits of biomarkers in plasma, as implemented in the exact RanksTests.

For box and whisker plots, the shaded box represents the first and third quartile of the population, and the whiskers extend to the 1.5 interquartile range. The black bar and shaded circles represent CD69 MFI median and mean, respectively.

## Results

### Patient characteristics

We initiated a study in which whole blood was collected from patients with RA for the measurement of PRT062607 activity in Syk-mediated ex vivo immune function assays. These data were then related to various parameters including disease severity, concomitant medications, and concentrations of serum proteins relevant to inflammation, with the specific goal of identifying variables that affect the activity of PRT062607 in modulating immune function. Thirty patients were enrolled in the study (two patients donated twice for a total of 32 samples). A broad distribution of disease severity was obtained, as measured by DAS28 ESR and DAS28 CRP scores. Concomitant medications included MTX (56%), prednisone (75%), and TNF antagonists (31%). A detailed breakdown of patient characteristics is provided in Table 1.

### Syk-independent mechanism(s) influence B-cell activation in whole blood from RA patients

**Figure 1:**
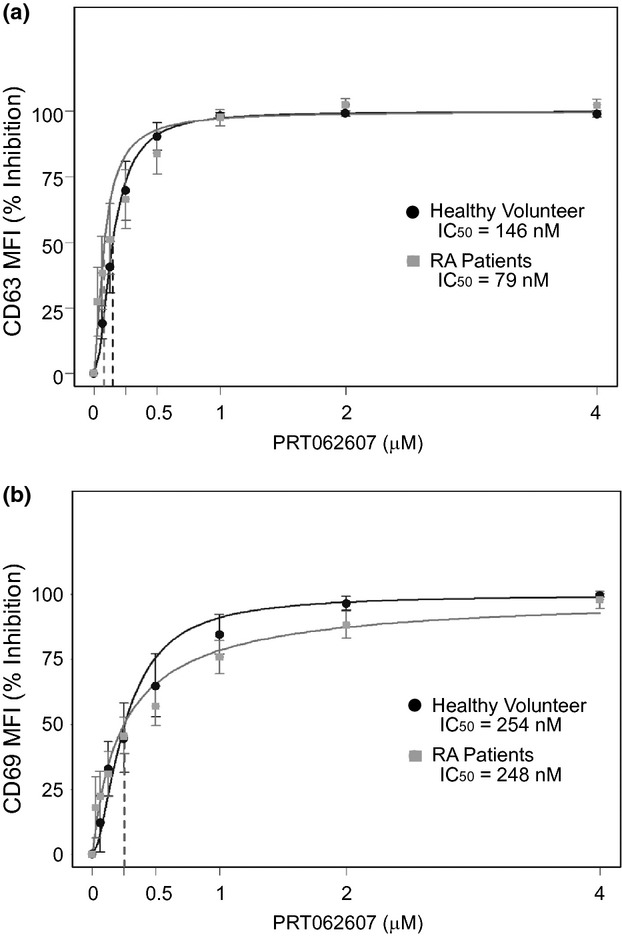
Syk-independent mechanism(s) influence BCR-mediated B-cell activation in whole blood from RA patients. The PRT062607 concentration-effect relationship in the basophil degranulation assay (A) and B-cell activation assay (B) is shown for healthy normal volunteers (*n* = 13 and 17, respectively) and in RA patients (*n* = 28 and 31, respectively). PRT062607 concentration is depicted on the *x*-axis in μmol/L, and the corresponding percent inhibition of immune cell activation on the *y*-axis. Data represent means ± SEM. The IC_50_ derived from each concentration-effect relationship is shown.

**Figure 2:**
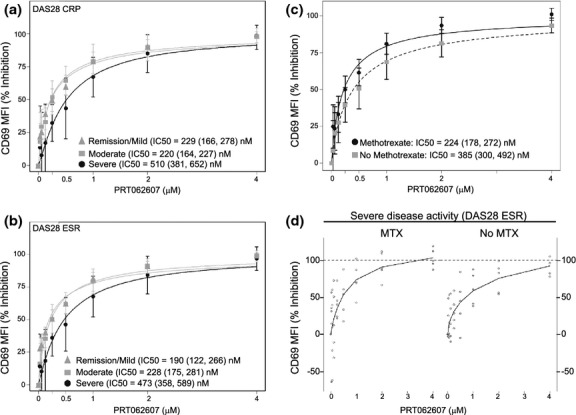
The dependency of BCR-mediated B-cell activation on Syk is affected by disease activity and treatment with MTX. DAS28-CRP (A), DAS28-ESR (B) scores were used to group patient data in three categories of disease activity; Remission/Mild (by DAS28-CRP *n* = 11, by DAS28-ESR *n* = 7), Moderate (by DAS28-CRP *n* = 13, by DAS28-ESR *n* = 15), and Severe (by DAS28-CRP *n* = 8, by DAS28-ESR *n* = 10). PRT062607 concentration (*x*-axis) by percent inhibition of B-cell activation (*y*-axis; mean ± SEM) is shown, along with the IC_50_ and 95% confidence interval. (C) The concentration-effect relationship was compared in RA patients that received (MTX; *n* = 18) or did not receive (No MTX; *n* = 14) stable MTX therapy. The IC_50_ and 95% confidence interval for each group are shown. Data are represented as mean ± SEM. (D) RA patients with severe activity as defined by DAS28-ESR scores were separated into two groups based on treatment with MTX. Raw data are shown (*n* = 5 per group) with a curvefit.

We employed two independent ex vivo immune function tests to explore the potency of Syk inhibition by PRT062607 in whole blood from RA patients, relative to healthy normal control (Coffey et al. 2011). In the first assay, basophils were stimulated with anti-IgE antibody to cross-link the FcεRI, initiating a Syk-dependent signaling pathway that leads to basophil degranulation (measured by upregulation of cell surface CD63). We observed no difference in the potency of PRT062607 to suppress basophil degranulation in healthy versus RA whole blood, indicating that Syk dependency for this immune response was unaffected by inflammation or concomitant medications (Fig. 1A). In the second assay, peripheral blood B cells were stimulated through the BCR to induce cellular activation (measured by upregulation of cell surface CD69). We observed that while the IC_50_ was unaffected between the two populations, the ability of the Syk inhibitor to achieve IC_75_ and greater was impaired in whole blood from RA patients, suggesting that Syk-independent mechanism(s) were influencing the ability of PRT062607 to suppress B-cell activation (Fig. 1B). To explore this phenomenon further, the RA population was divided into three groups, representing remission/mild, moderate, and severe disease activity as measured by DAS28 ESR or DAS28 CRP. Inhibition of BCR-mediated B-cell activation by PRT062607 was then compared among the groups (Figs. 2A and B). The remission/mild and moderate disease severity groups had comparable IC_50_′s with non-overlapping confidence intervals, and were also not different from healthy controls. In patients with severe disease, however, two observations were made. First, there was substantially more variability in the response to PRT062607, and second, the IC_50_ was increased from 190–229 nmol/L to 473–510 nmol/L. The altered Syk dependency for B-cell activation was therefore isolated to the severe inflammation group, suggesting that additional factors influencing B-cell function were involved.

### MTX uniquely restores PRT062607 inhibitory potency in suppression of BCR mediated B-cell activation

We next evaluated the effect of stable MTX therapy on the potency of PRT062607 in suppressing BCR-mediated B-cell activation in RA patients. Irrespective of the severity of disease activity, the population was separated into two groups; those on stable MTX therapy (*n* = 18) and those not receiving MTX (*n* = 14). Percent inhibition of B-cell activation across a range of PRT062607 concentrations was plotted (Fig. 2C). By comparing the two concentration-effect relationships, we observed that the activity of PRT062607 in MTX-treated patients (IC_50_ = 224 nmol/L) was similar to that of healthy controls, while for those patients not on MTX the IC_50_ (385 nmol/L) was higher. The confidence intervals between these two groups were nonoverlapping, and the effect was statistically significant by the Wilcoxon test. Furthermore, it was apparent that complete inhibition (defined as >80%) was more readily achieved by PRT062607 in the MTX-treated patients. Although limited by sample size, the same general observation was made in patients with severe inflammation, separated into two groups (*n* = 5 per group), those receiving MTX and those not. Raw data from this analysis are presented in Figure 2D. Importantly, when the patient population was grouped-based on prednisone or TNF inhibitor therapy, no impact on the potency of PRT062607 was observed (data not shown), indicating that MTX was unique in its ability to cooperate with PRT062607 to suppress B-cell function. No changes were observed in the percent of circulating B cells in the lymphocyte population among the various RA subgroups analyzed in the study (data not shown). Also, BCR/Syk signaling (Fig. S1A) was not affected by disease severity (Fig. S1B) or by MTX (Fig. S1C), suggesting that MTX affected the potency of PRT062607 inhibition of BCR-mediated functional responses by a Syk-independent mechanism.

### MTX treatment is associated with decreased serum cytokine concentrations

**Figure 3:**
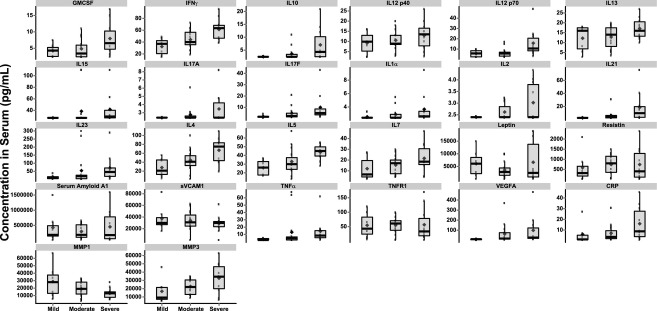
Serum cytokines and markers of inflammation change in accordance with disease severity in RA patients. Data depict serum protein concentration (pg/mL) as it relates to disease activity defined by DAS28-ESR as remission/mild (Mild), Moderate, and Severe. The shaded box represents the first and third quartile of the population, and the whiskers extend to the 1.5 interquartile range. The median is shown as the horizontal black bar and the mean by the closed circle. The specific serum protein measured is listed at the top of each figure.

MTX controls immune function in part by reducing cytokine burden (Cutolo et al. 2001; Wessels et al. 2008). We therefore utilized fresh frozen serum samples obtained from each of the RA patients to quantify concentrations of various cytokines and other serum markers of disease relevant to RA. As an initial analysis of this data, we sought to confirm the clinical observations and scoring of disease activity by assessing the relationship between disease activity and concentration of the serum proteins. Protein data were separated into three groups, representing remission/mild, moderate, and severe disease based on DAS28 ESR scores, and plotted against concentration on the *y*-axis as shown in Figure 3. Increased serum concentrations of several cytokines were observed in patients with severe disease, relative to mild or moderate. Most prominently these included granulocyte/monocyte colony-stimulating factor, interferon γ, IL10, IL2, IL4, and IL5. CRP and matrix metalloproteinase 3 were also elevated in the severe disease group. Correlation coefficients between all serum proteins measured, clinical observations, and DAS28 ESR and DAS28 CRP scores were also determined (Fig. S2). As expected, tender joint count, swollen joint count, and CRP strongly correlated with DAS scores (*R*^2^ > 0.7). The only additional serum proteins that achieved comparable correlation coefficients were IL2, IL4, and interferon γ.

**Figure 4:**
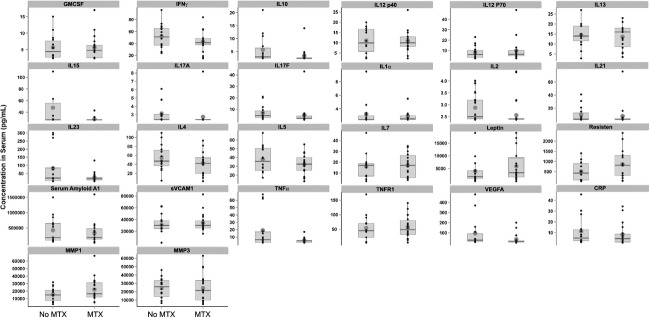
Treatment with MTX is associated with significant decreases in serum IL2 and IL17A. Serum cytokines and protein markers of inflammation were compared between RA patients on stable MTX therapy (MTX) or not receiving MTX (No MTX). Statistically significant differences between the two groups were determined by the Wilcoxon test (*P* ≤ 0.05). Raw data (black dots) are overlaid with the box and whisker plots that represent the first and third quartile of the population (shaded box), and the whiskers extend to the 1.5 interquartile range. The black bar represents the median and large shaded circle the mean. Serum concentration of each protein is plotted on the *y*-axis as pg/mL.

**Figure 5:**
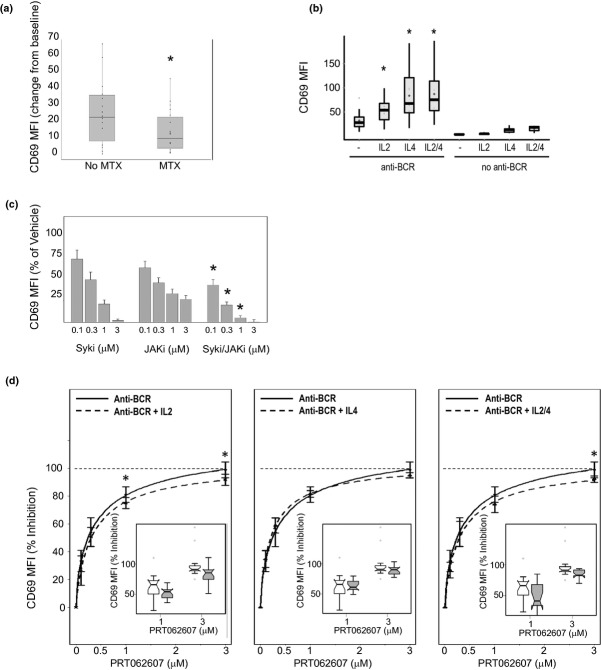
Cytokines and JAK/STAT signaling influence BCR-mediated B-cell activation. (A) Change from baseline in B-cell CD69 upregulation following BCR stimulation is compared between RA patients on stable MTX therapy (MTX) or not receiving MTX (No MTX). Raw data (block dots) are overlaid with box and whisker plots that represent the CD69 MFI on the *y*-axis. The shaded box represents the first and third quartile of the population, and the whiskers extend to the 1.5 interquartile range. The black bar represents the median and large shaded circle the mean. (B) The effect of costimulation of the BCR with IL2 or IL4 on B-cell activation is shown. B-cell CD69 MFI is plotted on the *y*-axis, and represented in the box and whisker plots. The stimulation conditions are shown on the *x*-axis. (C) The effect of Syk (Syki), JAK (JAKi), and combined Syk/JAK inhibition (Syki/JAKi) on B-cell activation is shown. CD69 MFI normalized to% of vehicle control is plotted on the *y*-axis (mean ± SEM), and the concentration of each inhibitor (0.1–3 μmol/L) is shown on the *x*-axis. The asterisks represent significant differences comparing combined Syk/JAK inhibition to Syk inhibition alone at matching concentrations. (D) The PRT062607 concentration-effect relationship in response to BCR stimulation alone (Anti-BCR) or costimulation of the BCR with IL2 (Anti-BCR + IL2; left panel), IL4 (Anti-BCR + IL4; center panel), or IL2 and IL4 (Anti-BCR + IL2/4; right panel) is shown. Percent inhibition of CD69 MFI relative to vehicle control is plotted on the *y*-axis, and concentration of PRT062607 in μmol/L on the *x*-axis. The dashed line across each panel represents the point of 100% inhibition, and asterisks represent statistical differences by Wilcoxon test (*P* < 0.05). The inset box and whisker plots depict the 1 and 3 μmol/L PRT062607 concentrations only.

We next determined the effect of MTX on serum concentrations of cytokines and markers of inflammation. Several of the serum proteins measured trended lower in patients on stable MTX, two of which were significantly decreased as determined by the Wilcoxon test, criteria set at *P* < 0.05. These were IL2 (*P* = 0.034) and IL17a (*P* = 0.027; Fig. 4). This effect was unique to MTX, as neither prednisone nor TNF inhibitors led to significant reductions in any of the serum proteins measured (data not shown). While MTX likely exerts immune modulation by multiple mechanisms, the reduction in IL2 was intriguing because this cytokine lowers the threshold for activation, differentiation, and clonal expansion of both B and T cells. In contrast, IL17 has no known role for directly modulating B-cell function, consistent with the observation that IL17a receptor expression is restricted to T and natural killer cells. Given the reduction in proinflammatory cytokine burden in MTX-treated patients, we predicted that B cells may be less responsive to BCR-mediated cellular activation in RA patients on stable MTX therapy. We tested this by comparing the extent of CD69 upregulation following BCR ligation in whole blood from RA patients untreated or treated with MTX (Fig. 5A). B cells from patients treated with MTX were less responsive to BCR-mediated cellular activation (Wilcoxon test, *P* < 0.05). These data suggest that by reducing cytokine burden, MTX may influence BCR mediated B-cell activation, and possibly the dependency on Syk for immune cell activation.

### Cytokines and JAK/STAT signaling influence BCR-mediated B-cell activation

Various cytokines, including IL2 and IL4 (Tsudo et al. 1984; Waldmann et al. 1984; Zubler et al. 1984; Muraguchi et al. 1985; Clark et al. 1989) have been shown to lower the threshold for BCR-mediated B-cell functional responses when added to cell suspensions. To confirm the involvement of cytokines in potentiating B-cell activation, we costimulated whole blood with IL2, IL4, and anti-BCR antibody to evaluate the effect on B-cell activation. As shown in Figure 5B, BCR ligation alone leads to upregulation of CD69. Costimulation of the BCR with IL2, IL4, or the two cytokines in combination dramatically enhanced the overall induction of B-cell activation (*P* < 0.05 for each costimulation condition relative to BCR ligation alone). IL2 stimulation alone was no different from the unstimulated control; whereas IL4 stimulation alone or in combination with IL2 had a minimal impact on B-cell activation, demonstrating that these cytokines primarily work in concert with signals originating from the BCR.

These data imply that cytokine-mediated JAK/STAT signaling may independently contribute to BCR/Syk-mediated B-cell activation. We tested this pharmacologically by evaluating B-cell activation in the presence of increasing concentrations of the Syk-selective inhibitor PRT062607, the JAK-selective inhibitor CP690,550 (Karaman et al. 2008) and the two inhibitors in combination (Fig. 5C). Results from these studies demonstrate the critical contribution JAK kinase(s) play in modulating B-cell activation in response to BCR ligation. As depicted, CP690,550 potently suppressed B-cell activation, although its effect was limited and it was unable to bring about full suppression of this functional response. By contrast, Syk inhibition alone by PRT062607 was able to fully suppress B-cell activation in a concentration-dependent manner. Of particular interest was the observation that when combined, dual suppression of both Syk and JAK kinases more potently inhibited B-cell functional responses relative to either agent alone (statistical significance indicated by asterisks). These data indicate that Syk and JAK contribute to the overall response of B cells to BCR ligation.

Finally, we evaluated the ability of IL2 and IL4 costimulations to influence the potency of PRT062607 in suppressing BCR-mediated B-cell activation. The potency of PRT062607 was compared in whole blood stimulated by BCR ligation alone, or in the presence of IL2 (Fig. 5D, left panel), IL4 (Fig. 5D, center panel), and IL2 plus IL4 (Fig. 5D, right panel). IL2 in isolation appeared only to have a subtle effect on PRT062607 potency against BCR-mediated B-cell activation, although the effect was significant (*P* < 0.05) at both the 1 and 3 μmol/L concentrations (data are re-plotted as box and whisker plots and subset within the overall curvefit). This result was recapitulated with the combination stimulation using IL2 plus IL4, but interestingly not with IL4 costimulation alone. We conclude from these experiments that cytokines and JAK/STAT signaling do influence B-cell functional responses, and that MTX may mitigate this influence by reducing proinflammatory cytokine burden. These data provide a rationale for the combined use of Syk inhibition and MTX for the treatment of autoimmune disease.

## Discussion

MTX is a widely used drug. There are several proposed mechanisms of action for MTX (reviewed by [Wessels et al. 2008]), including its ability to reduce proinflammatory cytokine burden by increasing extracellular concentrations of adenosine. Genetic evidence supporting this mechanism of action was recently reported using a mouse model of thioglycollate-mediated peritonitis. Treatment with MTX increased adenosine levels in the peritoneal exudates, and decreased leukocyte infiltration and levels of TNFα in the peritoneal space in wild-type and adenosine A3 receptor knockout mice, but not in adenosine A2 receptor knockout mice (Montesinos et al. 2006), demonstrating that the mechanism of anti-inflammatory activity of MTX requires adenosine and the A2 receptor. The anti-inflammatory activity of MTX in animal models is blocked by adenosine receptor antagonism (Cronstein et al. 1993). In RA patients, MTX treatment also results in increased serum concentrations of adenosine (Riksen et al. 2006). Hence, the ability of MTX to suppress cytokine responses appears to be important for its anti-inflammatory effects. Other cytokine modulating therapies such as antibodies against IL6 and the JAK family kinase inhibitor CP690,550 (tofacitinib) are also approved for use in RA patients (Coombs et al. 2010).

B cells have also emerged as a critical mediator of disease pathogenesis in RA (reviewed by [Panayi 2005]). Their contribution to inflammation may be threefold: (1) generation of a self-perpetuating auto-antibody response which leads to immune complex deposition within tissues, (2) BCR-mediated antigen uptake, presentation to, and activation of T cells, and (3) B-cell cytokine release. B cells are an important source of TNFα. Clonal expansion of B cells is observed in RA patients (Itoh et al. 2000), as is an activated phenotype represented by increased CD86 and decreased FcγRIIb expression (Catalan et al. 2010). B-cell depletion by anti-CD20 antibody (rituximab) has demonstrated efficacy in RA patients. These data indicate that B cells play an important role in the maintenance of this disease, and strategies to control B-cell function may therefore impact disease activity.

In recent years, genetic and pharmacological studies have shed additional light on the biological mechanisms underlying inflammatory processes. Of particular interest are signaling pathways that operate in immune cells which lead to such functional responses as clonal expansion, extravasation to sites of tissue injury and the release of mediators of inflammation and tissue damage. Syk appears prominently as a key regulator of immune function, controlling both innate and adaptive immune responses via the BCR, FcR, integrins, and others (Turner et al. 2000; Mocsai et al. 2002; Rogers et al. 2005). Syk is of particular interest as a target for modulation of B cells in RA in part because of the requirement for this kinase for BCR-derived signals that lead to activation and differentiation to memory B cells and antibody secreting plasma cells. Reconstitution of irradiated mice with Syk-deficient hematopoietic cells fail to mount inflammatory responses in the KBxN serum transfer-model (Jakus et al. 2010). The BCR is also critically involved in antigen uptake for presentation to T cells, which may contribute to the inflammatory process in RA. Syk is also required for signaling through the activating Fc receptors, but not through the inhibitory FcγRIIb. We recently reported on the ability of PRT062607 (also called P505-15) to suppress BCR-mediated signaling and cellular activation in healthy volunteer whole blood, and demonstrated dose-dependent reductions of inflammation in rodent models of RA (Coffey et al. 2011).

The data presented herein provide additional evidence for the ability of MTX to suppress serum cytokine levels in RA patients. It is well documented in the literature that cytokines can lower the threshold for B-cell activation in response to BCR ligation (Tsudo et al. 1984; Waldmann et al. 1984; Zubler et al. 1984; Muraguchi et al. 1985; Clark et al. 1989; Butcher and Cushley 1991; Braun et al. 2002). Our data support this, and demonstrate that cytokines and JAK/STAT signaling in general have a significant impact on B cell functional responses to BCR ligation. The potency of PRT062607 in suppressing BCR-mediated B-cell function was significantly enhanced by inclusion of tofacitinib (JAK1/3 inhibitor), and subtly reduced by inclusion of IL2. We conclude from these data that cytokines have the potential to exacerbate B-cell responses to antigen, and that MTX and PRT062607 likely affect distinct inflammatory mechanisms operative in RA to control B-cell function by dual suppression of cytokine and BCR signaling.

## Abbreviations

BCR: B-cell receptor
CRP: C-reactive protein
DAS: disease activity score
ESR: erythrocyte sedimentation rate
Fc: fragment crystallizable
IL: interleukin
JAK: janus kinase
MTX: methotrexate
RA: rheumatoid arthritis
Syk: spleen tyrosine kinase
TNF: tumor necrosis factor

## Supporting Information

**Figure S1.** Inflammation score and treatment with MTX do not significantly impact BCR-induced signaling responses in whole blood from RA patients. (A) Flow cytometry scatter plots from whole blood samples depicting the lymphocyte and B-cell gating strategies (first two panels) as well as the basal (No Stimulation; third panel) and BCR-induced (anti-BCR; fourth panel) tyrosine phosphorylation of Erk at amino acid position 204 (Erk Tyr204; a measure of Syk activity) and Syk at amino acid position 352 (Syk Tyr352; a measure of Lyn activity). (B and C) Basal (NS) and BCR-induced (anti-BCR) levels of Erk Tyr204 (measure of Syk activity) and Syk Tyr352 (measure of Lyn activity) are shown in whole blood from RA patients sub-grouped based on DAS28 ESR inflammation scores (B) and treatment with MTX (C). The *y*-axis depicts the MFI (mean + SEM).

**Figure S2.** Serum levels of CRP, IL2, IL4, and INFγ are strong correlates with DAS28-CRP/ESR. The heat maps represent correlations between DAS28-CRP (left) or DAS28-ESR (right) and serum proteins, tender joint count (TJC) and DAS28-CRP and DAS28-ESR scores as listed vertically on the map. Yellow indicates a strong positive correlation, while increased transition to blue indicates weaker positive correlations and negative correlations.
