# Supplementary figures and images for: Methotrexate and a spleen tyrosine kinase inhibitor cooperate to inhibit responses to peripheral blood B cells in rheumatoid arthritis
Source: Pharmacol Res Perspect. 2013 Dec 15;1(2):e00016. doi: 10.1002/prp2.16 (PMC4186432; doi:10.1002/prp2.16)

Supplemental Figure 1

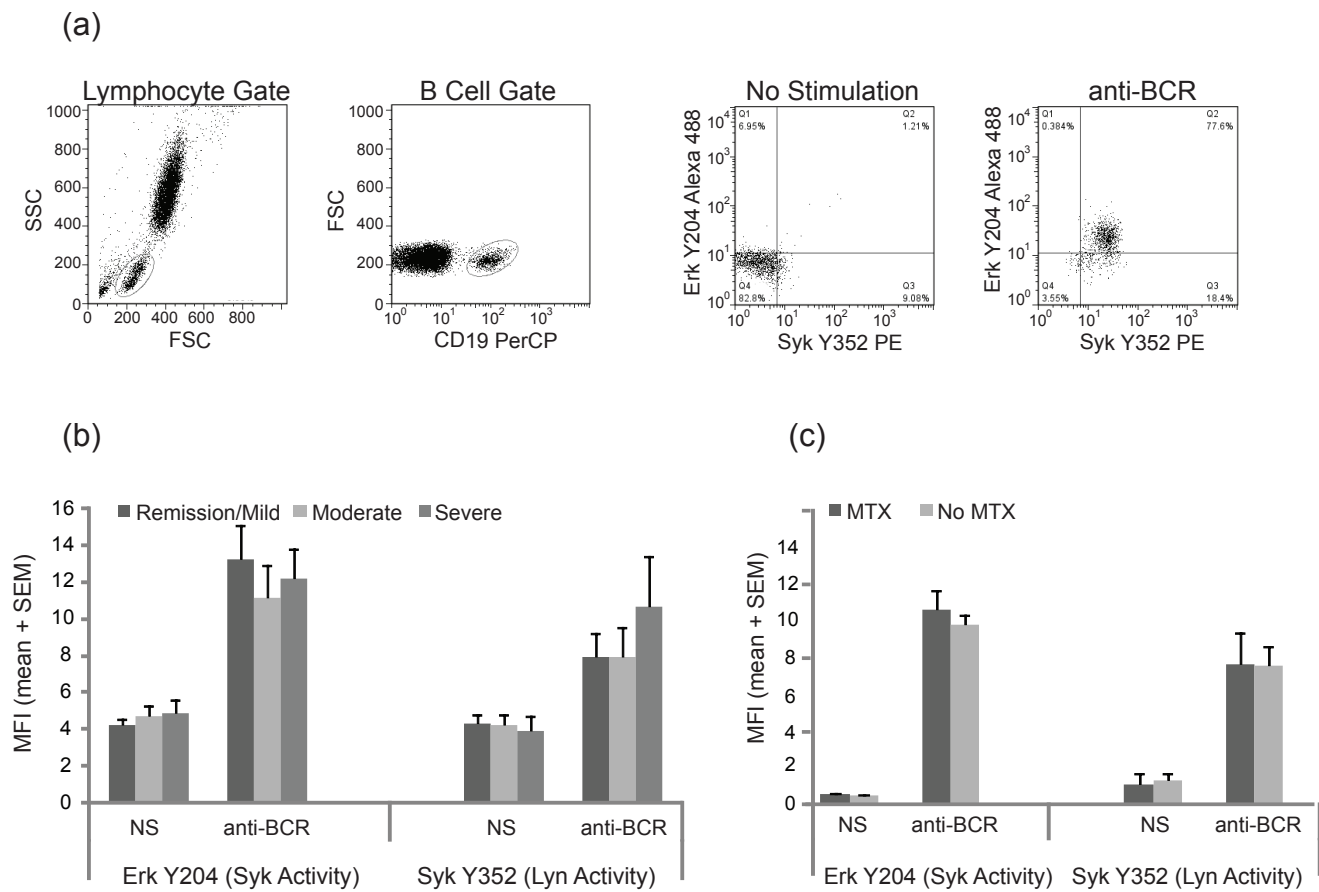

Supplement: Supplementary file 1 — Figure S1. Inflammation score and treatment with MTX do not significantly impact BCR-induced signaling responses in whole blood from RA patients. (A) Flow cytometry scatter plots from whole blood samples depicting the lymphocyte and B-cell gating strategies (first two panels) as well as the basal (No Stimulation; third panel) and BCR-induced (anti-BCR; fourth panel) tyrosine phosphorylation of Erk at amino acid position 204 (Erk Tyr204; a measure of Syk activity) and Syk at amino acid position 352 (Syk Tyr352; a measure of Lyn activity). (B and C) Basal (NS) and BCR-induced (anti-BCR) levels of Erk Tyr204 (measure of Syk activity) and Syk Tyr352 (measure of Lyn activity) are shown in whole blood from RA patients sub-grouped based on DAS28 ESR inflammation scores (B) and treatment with MTX (C). The y-axis depicts the MFI (mean + SEM). [file prp20001-e00016-SD1.pdf]

Supplemental Figure 2

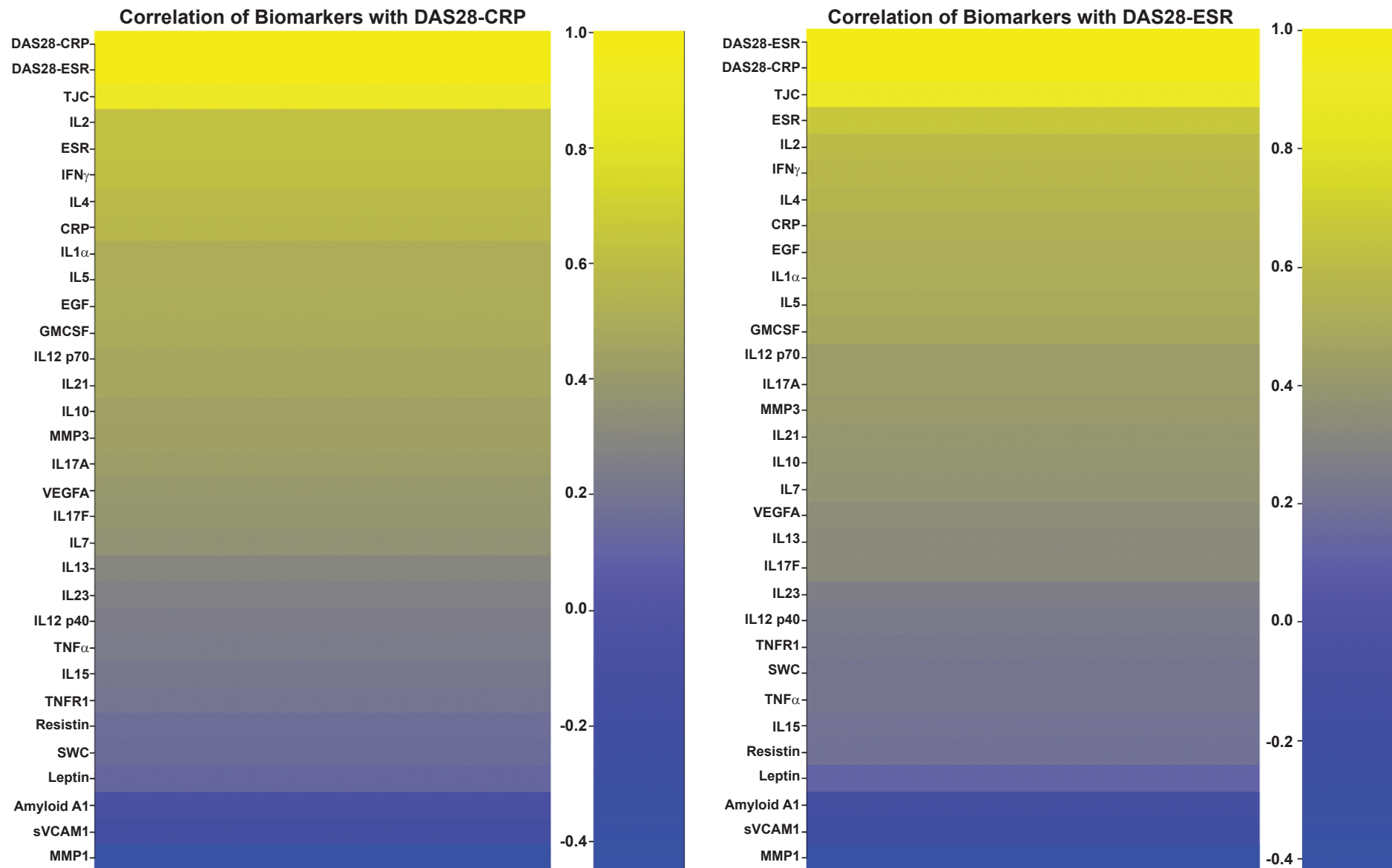

Supplement: Supplementary file 2 — Figure S2. Serum levels of CRP, IL2, IL4, and INFγ are strong correlates with DAS28-CRP/ESR. The heat maps represent correlations between DAS28-CRP (left) or DAS28-ESR (right) and serum proteins, tender joint count (TJC) and DAS28-CRP and DAS28-ESR scores as listed vertically on the map. Yellow indicates a strong positive correlation, while increased transition to blue indicates weaker positive correlations and negative correlations. [file prp20001-e00016-SD2.pdf]
